# Supplementary figures and images for: The promiscuous development of an unconventional Qa1b-restricted T cell population
Source: Front Immunol. 2023 Oct 31;14:1250316. doi: 10.3389/fimmu.2023.1250316 (PMC10644506; doi:10.3389/fimmu.2023.1250316)

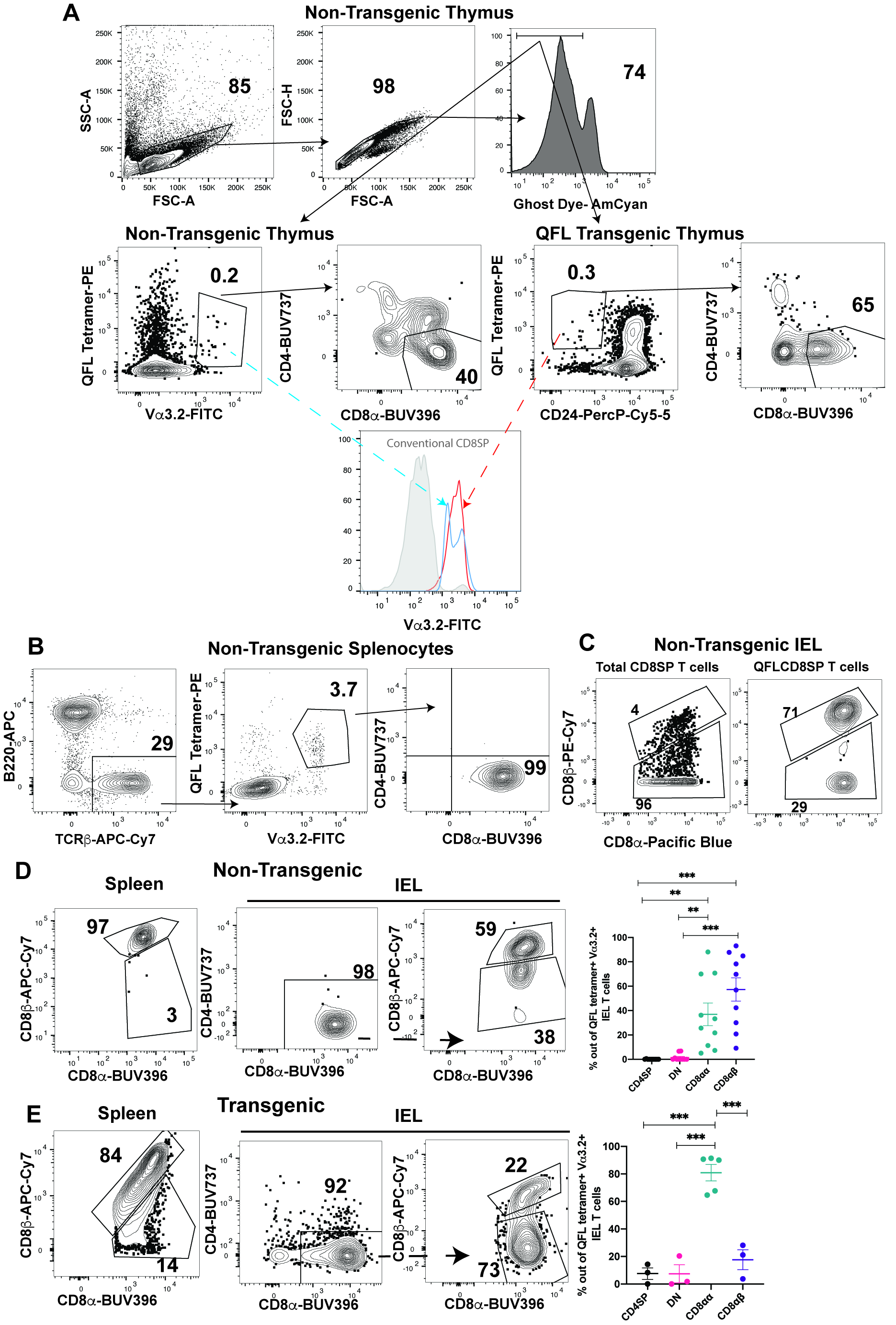

Supplement: Supplementary Figure 1 — Gating Strategy utilized to identify QFL T cells in distinct tissues. (A) Representative gating strategy employed to identify QFL CD8SP T cells in non-transgenic (tetramer enriched) and QFLTg thymocytes. Note that virtually all QFL tetramer+CD24- from QFLTg mice express Vα3.2 (lower histogram). (B) Representative plots showing the gating strategy to identify QFL CD8SP T cells in non-transgenic (tetramer enriched) and QFLTg spleen and IEL compartment of the small intestine. Example shown from Spleen of non-transgenic mice. (C) Representative flow plots illustrating gates identifying CD8αβ and CD8α+βlow populations within CD8SP T cells in the IEL compartment (Left) Total CD8SP T cells (Gated: B220-TCRβ+CD8α+CD4-) (Right) QFL CD8SP T cells (Gated: B220-TCRβ+QFL tetramer+Vα3.2+CD8α+CD4-) (D, E) (Left) Representative plots of CD8α and CD8β on QFL CD8SP splenocytes (Middle) Representative plots CD4, CD8α and CD8β of QFL T cells in the SI IEL compartment. (Right) Compiled data showing the % of indicated populations in QFL IEL compartment (Gated: TCRβ+QFL tetramer+Vα3.2+) (D) (n=10) and QFLTg (E) (n=5)mice. CD4SP (CD4+CD8α-) (Black dots), DN (CD4-CD8α-) (Magenta dots), CD8αα(CD4-CD8α+CD8β-) (Teal dots), CD8αβ (CD4-CD8α+CD8β+) (Purple dots). Error bars= Standard error of mean. Statistical Analysis: One way ANOVA followed by Tukey’s multiple comparison test was used to compare each sample to each other (P values are *<0.05,**<0.005,***<0.0005. Comparisons that are not statistically significant are not marked by a symbol. [file Image_1.tif]

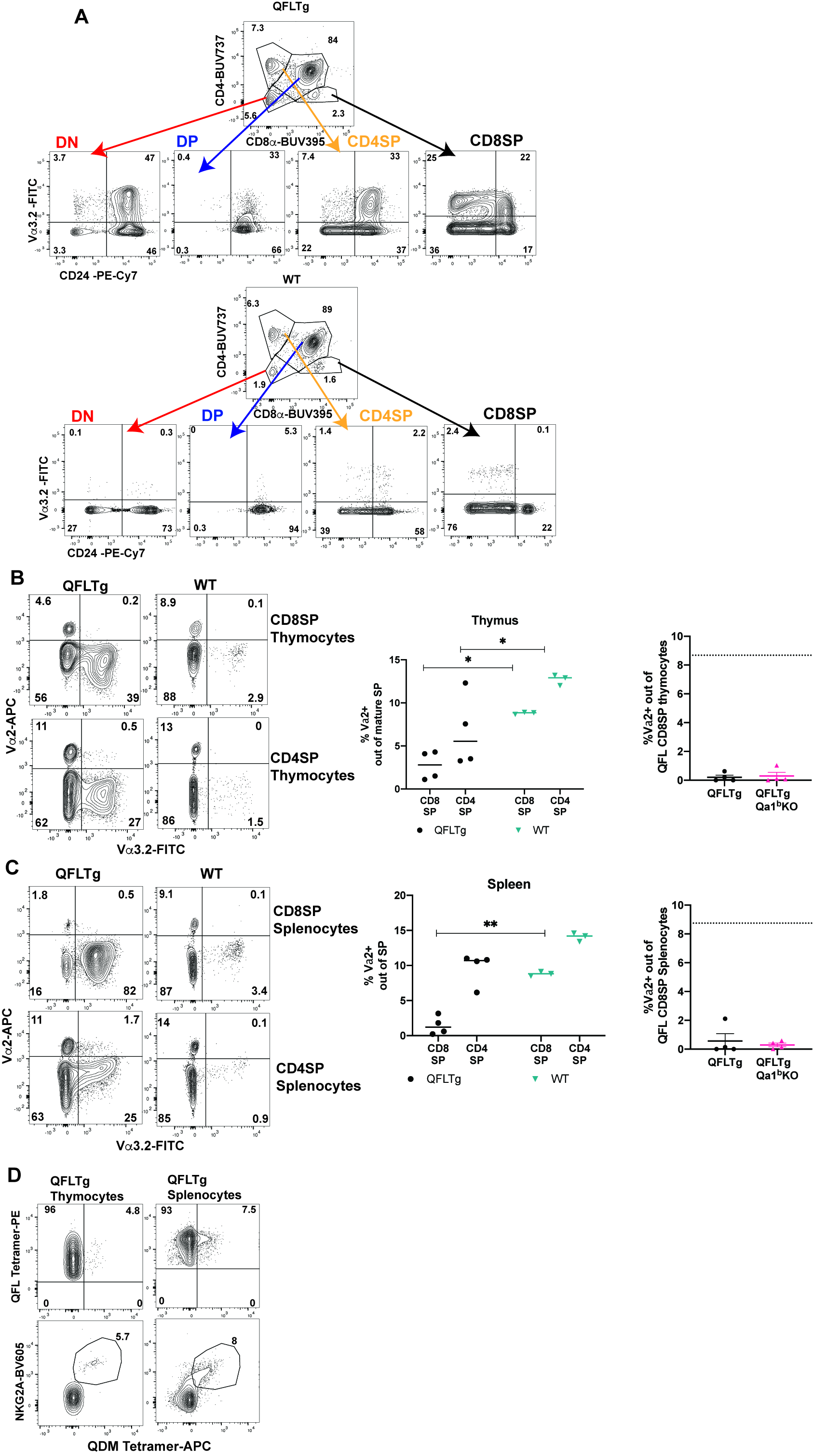

Supplement: Supplementary Figure 2 — Characterization of QFL TCR transgenic mice. (A) Representative flow plots of CD4 and CD8α expression on thymocytes from QFLTg mice (Gated: Live) or Vα3.2 and CD24 expression of indicated thymocyte populations. Data from wild type mice is show below for comparison. (B) Representative flow plots of Vα2 and Vα3.2 expression in CD8SP (Gated: CD8α+CD4-CD24-) and CD4SP (Gated: CD8α-CD4+CD24-) thymocytes from QFLTg and WT mice. Central dot plots show compiled data of Vα2 expression on SP thymocytes from QFLTg (n=4) and WT (n=3) mice. Right most plots show compiled data of Vα2 expression out of QFL CD8SP (Gated: QFL tetramer+CD24-CD8α+CD4-) thymocytes from QFLTg (n=4) and QFLTgQa1bKO (n=4) mice. Dotted line indicates average value for mature CD8SP thymocytes from wild type mice. Each dot represents an individual mouse. (C) Representative flow plots of Vα2 and Vα3.2 expression in CD8SP and CD4SP splenocytes from QFLTg and WT mice. Central dot plots show compiled data of Vα2 expression on SP splenocytes from QFLTg (n=4) and WT (n=3) mice [Gated: TCRβ+B220-CD8α+CD4- (CD8SP) or TCRβ+B220-CD8α+CD4- (CD4SP)]. Right most plots show compiled data of Vα2 expression out of QFL CD8SP (Gated: TCRβ+B220-QFL tetramer+CD8α+CD4-) splenocytes from QFLTg (n=4) and QFLTgQa1bKO (n=4) mice. Dotted line indicates average value for mature CD8SP splenocytes from wild type mice. Each dot represents an individual mouse. (D) Representative flow plots of (Top) QFL tetramer and QDM tetramer and (Bottom) NKG2A and QDM tetramer on QFLTg thymocytes (Gated: QFL tetramer+CD24-) and QFLTg splenocytes (Gated: TCRβ+B220-QFL tetramer+). Error bars= Standard error of mean. Statistical analysis: Two-way ANOVA followed by Tukey’s multiple comparison test comparing experimental samples to each other in their respective genetic background (B, C). Student’s t test comparing QFLTg and QFLTgQa1bKO samples to each other (B, C). P values are * <0.05, ** <0.005, ***<0.0005. Comparisons that are not statistically signific [file Image_2.tif]

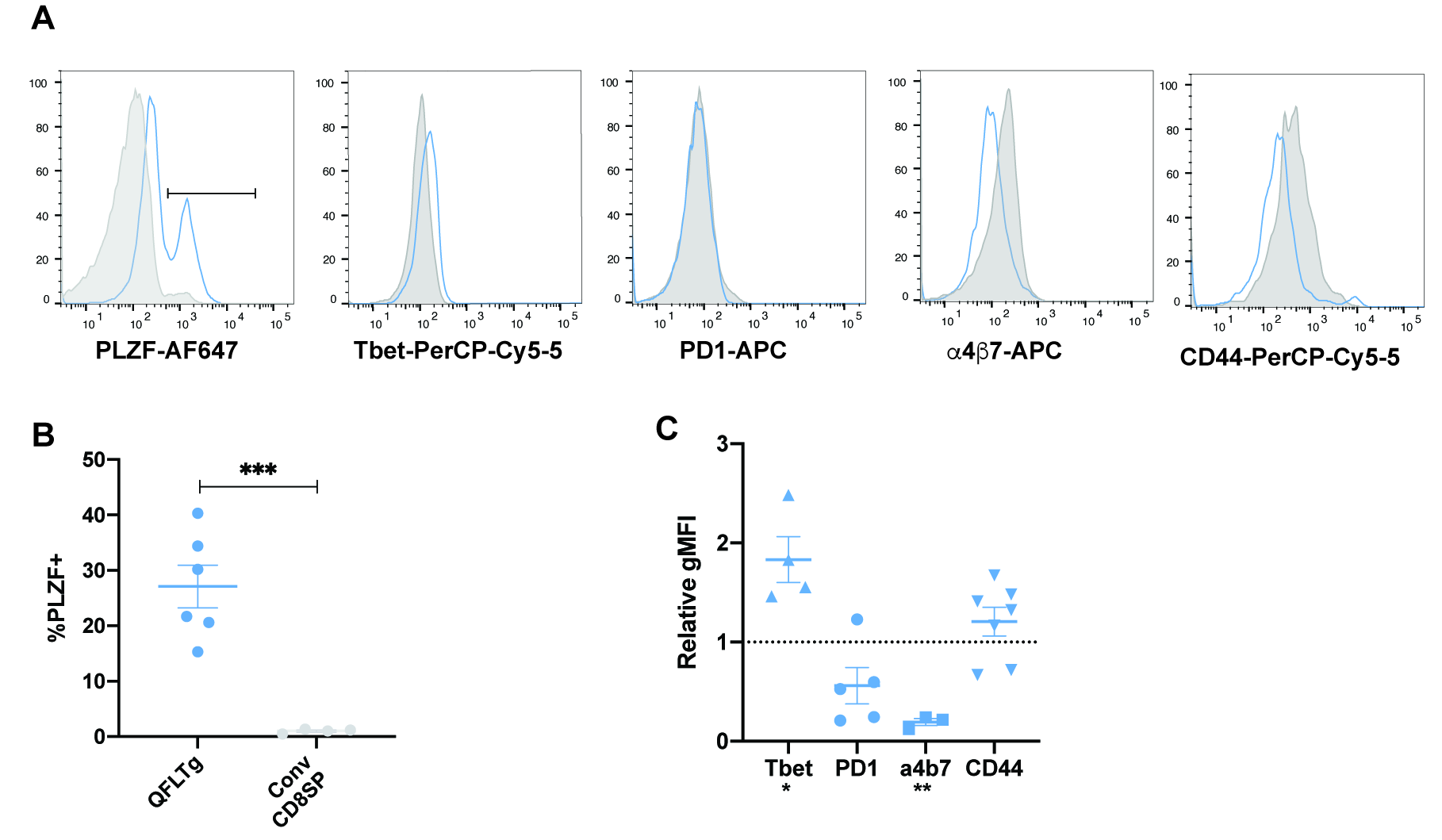

Supplement: Supplementary Figure 3 — Characterization of QFL CD8SP thymocytes. (A) Representative histograms of PLZF (n=6), Tbet (n=4), PD1 (n=5), α4β7(n=4) and CD44 (n=6) expression in QFL CD8SP thymocytes (Light Blue curve/dots) (Gated: QFL tetramer+CD24-CD8α+CD4-). Conventional CD8SP thymocytes (Grey histogram/dots) (Gated: CD8α+CD4-) are shown for comparison. (B) Percentage of PLZF+ out of QFL CD8SP thymocytes or conventional CD8SP (Conv CD8SP) thymocytes. (C) gMFI of Tbet, PD1, α4β7 and CD44 of QFL CD8SP thymocytes normalized to the gMFI of conventional CD8SP thymocytes(Dotted line). Error bars= Standard error of mean. Statistical analysis: Student’s t test was used to compare experimental samples to each other (B). One-sample t test was used to compare experimental samples to the control used for normalization (C): shown below experimental label. P values are * <0.05, **<0.005, ***<0.0005. Comparisons that are not statistically significant are not marked by a symbol. [file Image_3.tif]

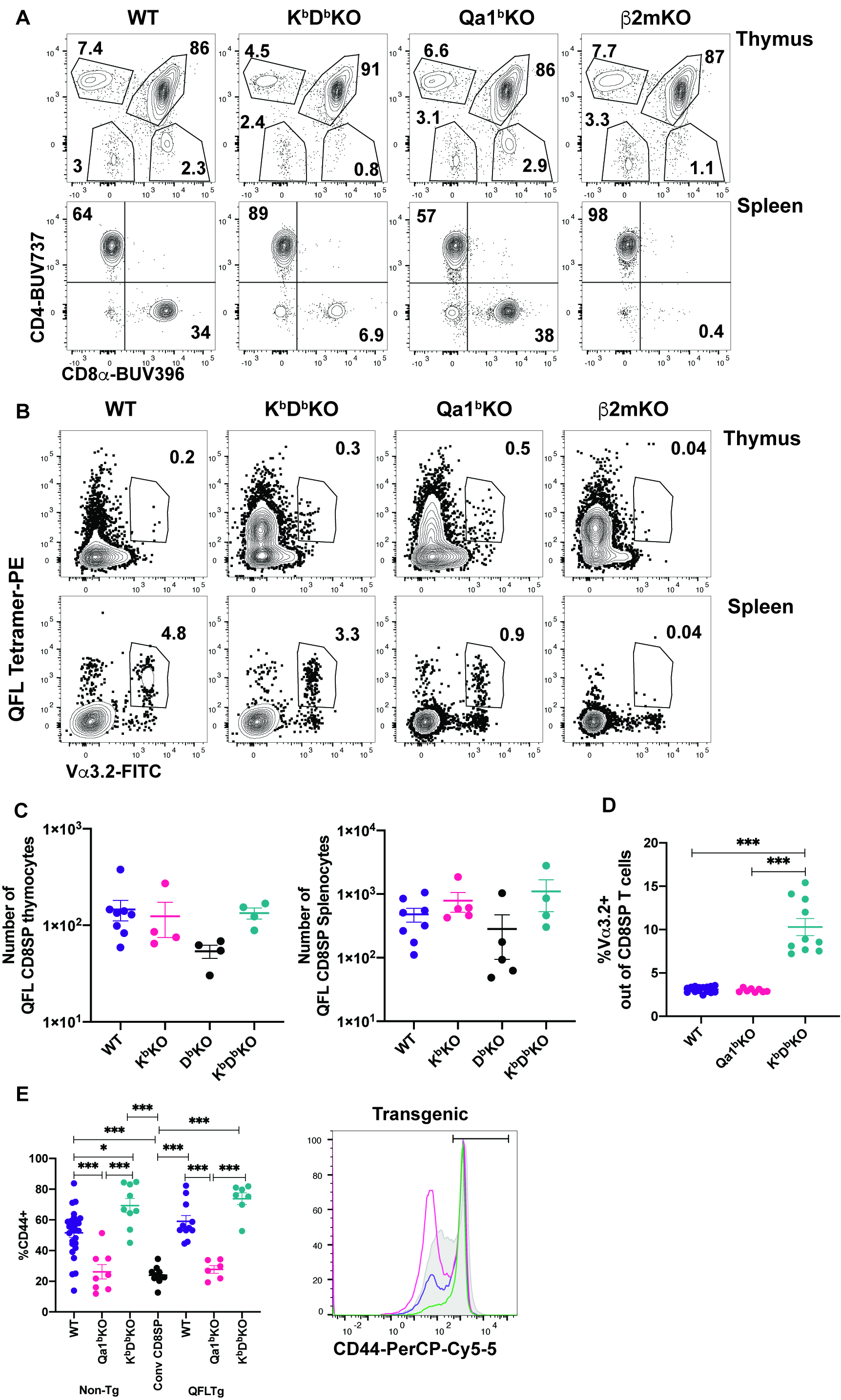

Supplement: Supplementary Figure 4 — Phenotype of KbDbKO and Qa1bKO thymus and spleen. (A) Representative flow plots of CD4 and CD8α expression of unenriched WT, KbDbKO, Qa1bKO and β2mKO thymocytes (Gated: Live cells) and splenocytes (Gated: TCRβ+). (B) Representative flow plots of QFL tetramer and Vα3.2 TCR expression on tetramer enriched thymocytes (Gated: Live) and splenocytes (Gated: TCRβ+) of B6, KbDbKO, Qa1bKO and β2mKO mice. (C) Number of QFL T cells in thymi (tetramer enriched and gated: QFL tetramer+Vα3.2+CD8α+CD4-) or spleen (tetramer enriched and gated: TCRβ+QFL tetramer+Vα3.2+ CD8α+CD4-) of non-transgenic mice of the indicated genotype. Thymus (WT (purple) n=9, KbKO (magenta) n=4, DbKO (black) n=4 KbDbKO (teal) n=4) Spleen (WT n=8, KbKO=5, DbKO=5 KbDbKO n=4). (D) Frequency of Vα3.2+ cells out of CD8SP splenocytes (Gated: TCRβ+B220-CD8α+CD4-) from the indicated mouse strains (WT (purple) n=16, Qa1bKO (magenta) n=8, KbDbKO (teal) n=10). (E) Representative histogram and compiled data of CD44 expression on QFLCD8SP T cells (Gated: TCRβ+QFL tetramer+Vα3.2+CD8α+) in Non-Transgenic (WT (purple) n=32, Qa1bKO magenta) n=8 and KbDbKO (teal) n=9), QFL Transgenic(WT n=11, Qa1bKO n=6 and KbDbKO n=7) and conventional CD8SP (conv CD8SP, black) (n=10) (Gated: TCRβ+CD8α+CD4-) from unenriched non-transgenic spleens. Error bars= Standard error of mean. Statistical analyses: One way ANOVA followed by Tukey’s multiple comparison test comparing each experimental sample to each other (C–E). P values are *<0.05, **<0.005, ***<0.0005. Comparisons that are not statistically significant are not marked by a symbol. [file Image_4.tif]

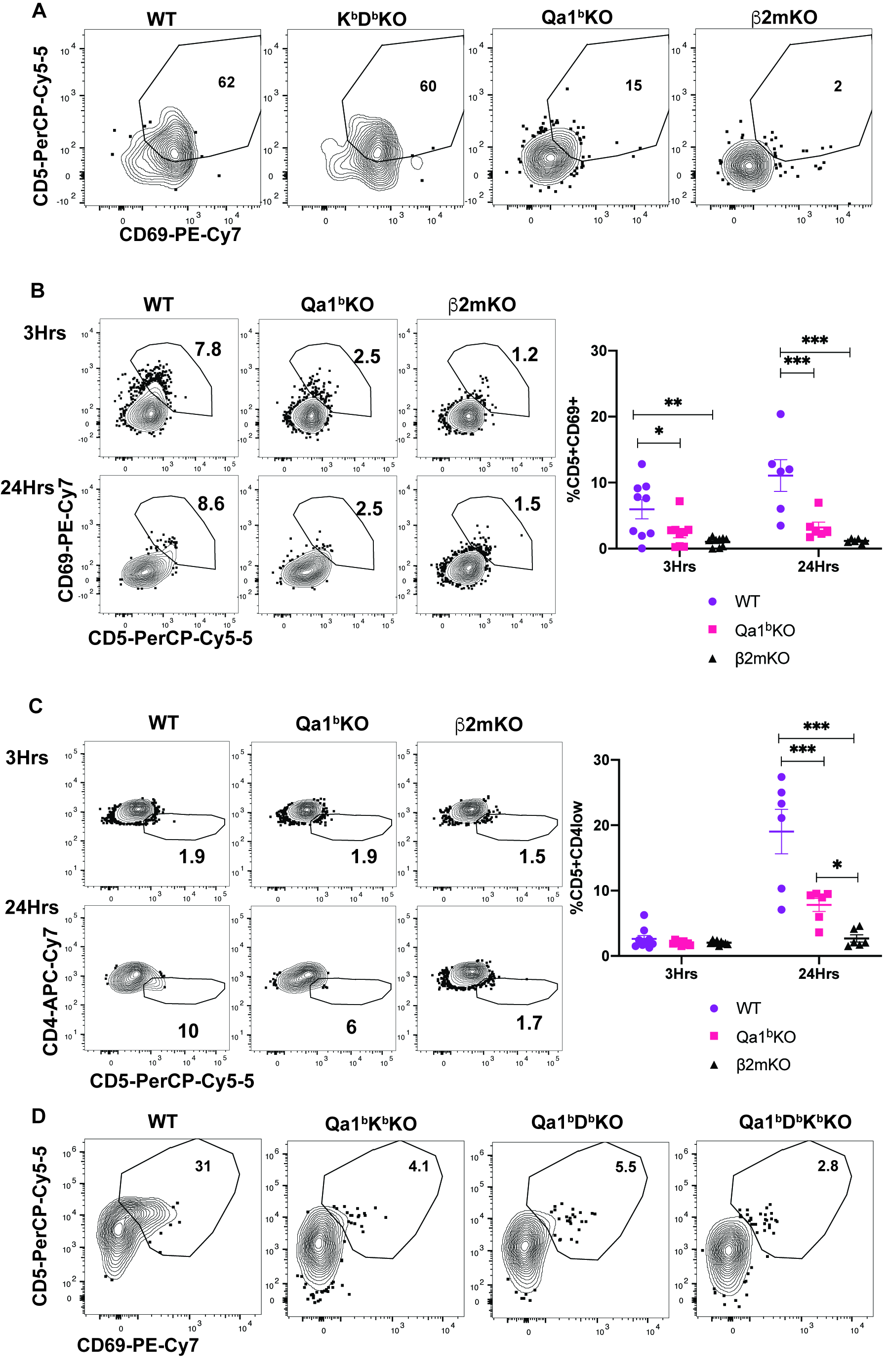

Supplement: Supplementary Figure 5 — QFL thymocyte stimulation in-vitro. (A) Representative flow cytometry plots of CD5 and CD69 expression on preQFLTg thymocytes (from QFLTg β2mKO mice) (Gated: QFL tetramer+Vα3.2+CD4+CD8α+) after 24 hours of co-culture with Bone Marrow Derived Dendritic cells (BMDC) from the indicated mouse strains. (B, C) PreQFLTg thymocytes were overlaid onto thymic tissue slices from the indicated mouse strains. Representative flow cytometry plots of (B) CD5 and CD69 expression or (C) CD5 and CD4 expression on QFL DP thymocytes (Gated: QFL tetramer+Vα3.2+CD4+CD8α+) after 3 (n=5 for all conditions) and 24 (n=5 for all conditions) hours of co-culture. Dot plots show compiled data of two experiments, with each dot representing a sample from an individual thymic slice. (D) Representative flow cytometry plots of CD5 and CD69 expression on preQFLTg DP thymocytes that were co-cultured for 24Hrs with either parental (WT) DC2.4 cells or DC2.4 cells in which genes encoding the indicated MHC molecules were knocked out using CRISPR/Cas9 editing (Gated: QFL tetramer+Vα3.2+CD4+CD8α+). Error bars= Standard error of mean. Statistical analysis: Two-way ANOVA followed by Tukey’s multiple comparison test comparing samples to each other in their respective time point (B, C). P values are *<0.05, ** <0.005, ***<0.0005. Comparisons that are not statistically significant are not marked by a symbol. [file Image_5.tif]

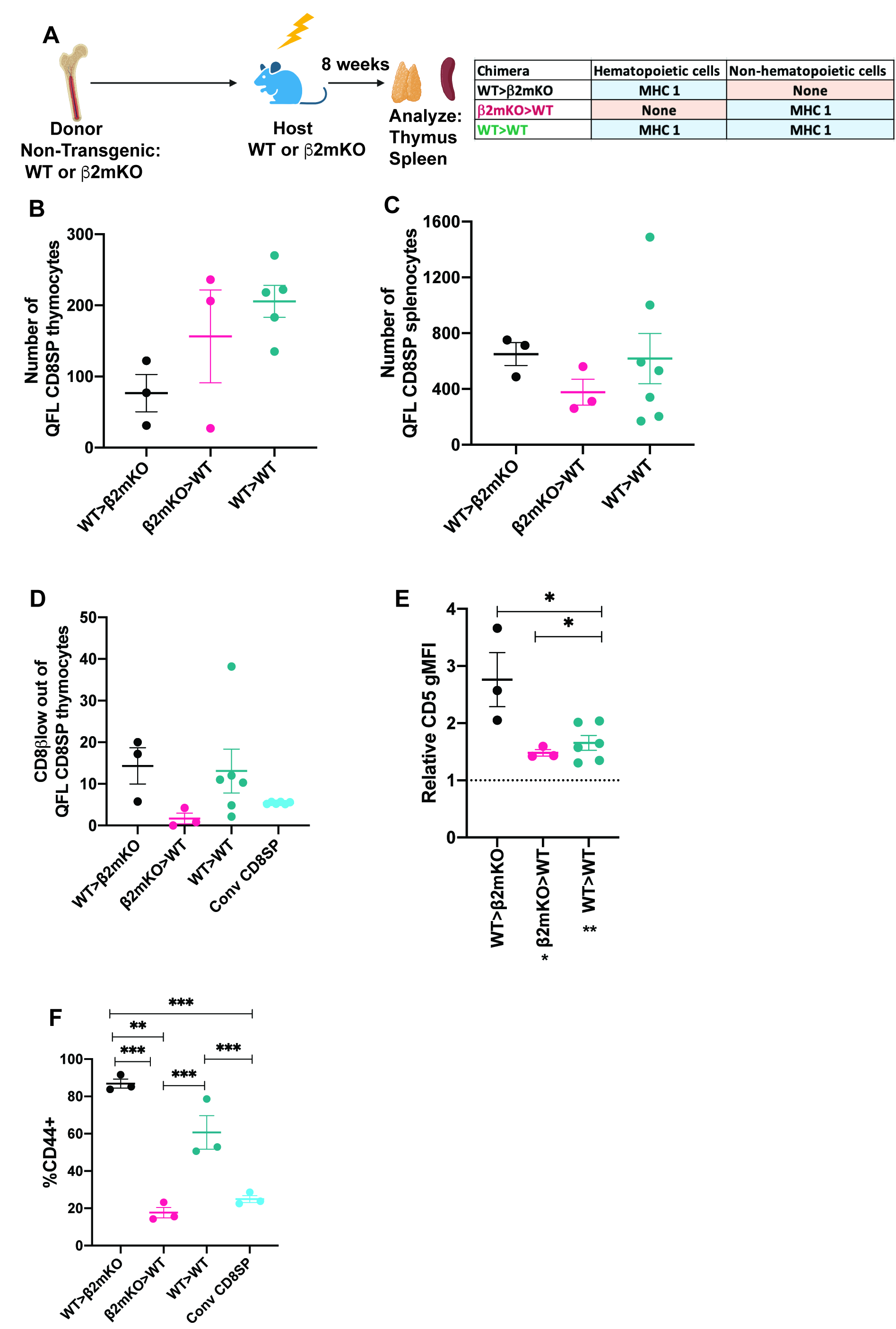

Supplement: Supplementary Figure 6 — Requirement for hematopoietic cells versus non-hematopoietic cell MHC I expression in QFL T cell development in non-transgenic mice. (A) Diagram of experimental design. Non-transgenic WT or β2mKO mice were used as bone marrow donors to reconstitute irradiated β2mKO or wild type hosts in order to restrict MHC I expression to hematopoietic or non-hematopoietic cells. (B, C) Absolute numbers of QFL CD8SP T cells in (B) thymus (tetramer enriched and gated: QFL tetramer+Vα3.2+CD8α+) and (C) spleens (tetramer enriched and gated: TCRβ+QFL tetramer+Vα3.2+CD8α+) from the indicated chimeric mice. WT>β2mKO (black dots, n=3), β2mKO>WT (magenta dots, n=3) and WT>WT (teal dots, n=5). (D) Downregulation of CD8β of QFL CD8SP thymocytes of the indicated chimeric mice (WT>β2mKO (black) n=10, β2mKO>WT (magenta) n=10, WT>WT (teal) n=5). Conventional CD8SP (Conv CD8SP) (light blue, n=3) (Gated: CD8α+CD4-) from unenriched non-transgenic mice shown for comparison. (E) Relative CD5 expression on QFL CD8SP thymocytes of the indicated chimeric mice. Graph shows gMFI of CD5 expression of QFL thymocytes normalized to the gMFI for conventional CD8SP (conv CD8SP) analyzed in the same experiment (WT>β2mKO n=3, β2mKO>WT n=3, WT>WT n=6). (F) Quantification of CD44 expression of QFL CD8SP T cells (Gated: TCRβ+QFL tetramer+Vα3.2+CD8α+CD4-) from tetramer enriched non-transgenic splenocytes from: WT>β2mKO (n=3), β2mKO>WT(n=3), WT>WT (n=3) chimeric spleens. For comparison conventional CD8SP (Conv CD8SP) (light blue) (n=3) (Gated: TCRβ+CD8α+) from unenriched non-transgenic mice. Error bars= Standard error of mean. Statistical analysis: One way ANOVA followed by Tukey’s multiple comparison test comparing each experimental sample to each other (B–F); shown above dots. One-sample t test was used to compare experimental samples to the control used for normalization (E); shown below label. P values are * <0.05, **<0.005, ***<0.0005. Comparisons that are not statistically significant are not marked by a symbol [file Image_6.tif]
